# Effects of Copper Oxide Nanoparticles on the Growth of Rice (*Oryza Sativa* L.) Seedlings and the Relevant Physiological Responses

**DOI:** 10.3390/ijerph17041260

**Published:** 2020-02-15

**Authors:** Zhongzhou Yang, Yifan Xiao, Tongtong Jiao, Yang Zhang, Jing Chen, Ying Gao

**Affiliations:** 1 College of Life Science, Northeast Normal University, Changchun 130024, China; yangzz121@nenu.edu.cn; 2 College of Life Science, Jilin Agricultural University, Changchun 130118, China; xyf98331@163.com (Y.X.); jtt1163933169@163.com (T.J.); zy18304315901@163.com (Y.Z.)

**Keywords:** *Oryza sativa*, CuO nanoparticles, membrane damage, oxidative stress, chlorophyll, carotenoids, gene expression

## Abstract

Rice (*Oryza sativa* L.), a major staple food for billions of people, was assessed for its phytotoxicity of copper oxide nanoparticle (CuO NPs, size < 50 nm). Under hydroponic condition, seven days of exposure to 62.5, 125, and 250 mg/L CuO NPs significantly suppressed the growth rate of rice seedlings compared to both the control and the treatment of supernatant from 250 mg/L CuO NP suspensions. In addition, physiological indexes associated with antioxidants, including membrane damage and antioxidant enzyme activity, were also detected. Treatment with 250 mg/L CuO NPs significantly increased malondialdehyde (MDA) content and electrical conductivity of rice shoots by 83.4% and 67.0%, respectively. The activity of both catalase and superoxide dismutase decreased in rice leaves treated with CuO NPs at the concentration of 250 mg/L, while the activity of the superoxide dismutase significantly increased by 1.66 times in rice roots exposed to 125 mg/L CuO NPs. The chlorophyll, including chlorophyll *a* and chlorophyll *b*, and carotenoid content in rice leaves decreased with CuO NP exposure. Finally, to explain potential molecular mechanisms of chlorophyll variations, the expression of four related genes, namely, *Magnesium chelatase D subunit, Chlorophyll synthase*, *Magnesium-protoporphyrin IX methyltransferase*, and *Chlorophyllide a oxygenase,* were quantified by qRT-PCR. Overall, CuO NPs, especially at 250 mg/L concentration, could affect the growth and development of rice seedlings, probably through oxidative damage and disturbance of chlorophyll and carotenoid synthesis.

## 1. Introduction

Engineered nanoparticles (ENPs) are particles with sizes ranging from 1 to 100 nm [1]. Due to their particular properties, they have been utilized for various purposes, such as in biomedicine, agriculture, and industries [2,3]. However, the extensive utilization of ENPs has resulted in their inevitable and irreversible release into the environment [4]. With ENPs expected to become more widely used, their effect on the environment and organisms has been an increasing cause of concern [5,6,7,8]. As plants interact with the atmosphere, soil, and water, they might be directly contaminated by ENPs. Thus, frequent assessment of the phytotoxicity of ENPs is considered to be of pivotal significance in environmental protection [9,10,11,12].

Among the various types of ENPs, copper oxide nanoparticles (CuO NPs) are extensively applied in various fields, such as high-temperature superconductors [13], batteries [14], gas sensors [15], lubricating oil additives [16], contamination remover [17], and catalysis [18]. It is conservatively estimated that, from 2020 to 2025, the world will consume at least 200,000–830,000 kg of CuO NPs every year [19]. The high demand and application of CuO NPs will greatly increase the possibility of their release into the environment and bioaccumulation into the food chain through plants, causing potential harm to human health [20,21]. Therefore, it is particularly important to evaluate the phytotoxicity of CuO NPs.

CuO NPs have been reported to exhibit phytotoxicity by causing a range of physiological effects, and they are likely to have different toxic effects on different plants at different target concentrations [9,22]. For example, copper oxide nanoparticles could inhibit *Brassica juncea L.* root and shoot growth in a dose-dependent manner [23]. In two studies, 500 mg/L copper oxide nanoparticles was shown to inhibit maize growth [24], while ˃10 mg/L copper oxide nanoparticles significantly inhibited cotton biomass accumulation [25]. It has also been reported that CuO NPs can exert negative influence on *Pisum sativum* [26], *Schoenoplectus tabernaemontani* [27], *Hordeum vulgare* [28], *Landoltia punctate* [29], and *Oryza sativa* (rice) [30,31]. CuO NPs were also found to inhibit root elongation of freshly germinated maize and rice seedlings, even at a low concentration of 25 mg/L [32]. The phytotoxicity of CuO NPs is usually assessed by measuring the phenotypic indexes of plant growth and development, e.g., germination rates, root elongation, and biomass accumulation [9,33,34]. However, compared to other nanoparticles, published papers focusing on the physiological and molecular changes caused by CuO NPs are still limited.

As a vital staple food in our daily lives, the safe production of rice is of critical importance. The phytotoxicity of CuO NPs in rice has been reported, and two main physiological factors are responsible for the phytotoxicity of CuO NPs: oxidative damage and impaired photosynthesis. For example, Shaw and Hossain [30] reported that CuO (<50 nm) at 0.5, 1.0, and 1.5 mM could reduce germination of rice seeds and viability of root cells but increase H_2_O_2_ and malondialdehyde (MDA) levels as well as ascorbate peroxidase (APX) and glutathione reductase (GR) activity. Furthermore, CuO NPs at 1000 mg/L could reduce photosynthetic rate, transpiration rate, and photosynthetic pigment content and increase the content of MDA and proline in rice [31]. However, the molecular mechanisms by which CuO NPs cause the phytotoxicity are still unclear. More studies are needed to improve our understanding of the mechanisms.

The aims of this study were to (i) investigate the phenotypic changes caused by short-time exposure of CuO NPs to rice seedlings at early stage under hydroponic culture; (ii) examine the changes of physiological factors, e.g., oxidative damage and impaired photosynthesis, caused by CuO NPs in seedlings; and (iii) evaluate the potential mechanisms of the decreased chlorophyll in rice leaves exposed to CuO NPs at the molecular level.

## 2. Materials and Methods

### 2.1. CuO NP Characterization

CuO NPs (<50 nm, Product #544868) were purchased from Sigma Aldrich Chemical Co. (St. Louis, MO, USA). The morphology of CuO NPs was elliptic or spherical with size ranging from 40 to 80 nm as observed by transmission electron microscope (TEM). The purity of CuO NPs was greater than 97%, and the pH of CuO NP suspensions (2000 mg/L) was 6.36 ± 0.02. Details of the TEM figure and the zeta potential of CuO NP suspensions have been published previously [21].

### 2.2. Culture of Rice Plants

Seeds of rice (*Oryza sativa* spp. *japonica*) were harvested in 2018 and stored in our laboratory. Their germination rate was proved to be higher than 90% before experiments. The seeds were first sterilized using sodium hypochlorite (10%) for 10 min, then rinsed with sterilized deionized (DI) water five times to remove the remaining disinfectants, and germinated in a 100 mm × 15 mm Petri dish containing 5 mL sterilized DI water in a growth chamber in the dark at 25 °C. After four days, 12 germinated seeds were transplanted to a 200 mL beaker with 200 mL Yoshida nutrient solution (pH 5.5) [35]. All the rice seedlings were grown in a chamber under the following conditions: 60%–70% relative humidity, 25/20 °C day/night temperature,14 h photoperiod, and a light intensity of 16,500 lx. In the next 10 days, the Yoshida nutrient solution in the 200 mL beaker was renewed every 2 days.

Before treatment, CuO NP suspensions were diluted in different contents of Yoshida nutrient solution (pH 5.5) at the concentration of 0, 62.5, 125, and 250 mg/L and sonicated using an ultrasonic vibration (100 W, 40 KHz) for 45 min. The supernatant from 250 mg/L CuO NP suspensions (S250) was obtained by sonicating (45 min) in Yoshida nutrient solution (pH 5.5), centrifuging for 30 min at 15,000 rpm, and then filtering through 0.22 μm filters. The 14-day-old rice seedlings were subjected to 0, 62.5, 125, and 250 mg/L CuO NPs and the supernatant from 250 mg/L CuO NP suspensions and renewed every 2 days. All the experiments were conducted with three biological replicates. After another 7 days of exposure, the 21-day-old seedlings from every beaker were harvested and washed thoroughly with DI water. The roots and leaves of the rice seedlings were separated, and their fresh weights (FW) were recorded.

### 2.3. Integrity of the Cell Membrane System of Rice

To evaluate the integrity of cell membrane, the lipid peroxidation level and ion leakage in rice roots and leaves were quantified. The lipid peroxidation was examined by the MDA method described by Heath and Packer [36] with minor modifications. Briefly, 0.5 g fresh tissue of rice roots and leaves was grounded in 0.1% (*w*/*v*) trichloroacetic acid (TCA). After being centrifuged for 20 min at 10,000 g, 20% TCA (1 mL) with 0.5% thiobarbituric acid (TBA) was added to 1 mL of the supernatant. After the addition of 100 μL 4% butylated hydroxyltoluene (BHT, in ethanol), the mixture was immediately heated in a water bath at 95 °C for 30 min, then quickly cooled and centrifuged for 15 min at 10,000 g. The supernatant was taken out to measure the absorbance at 532 and 600 nm with a Beckman Du-640 spectrometer (Beckman Coulter, Brea, CA). Then, 0.25% TBA in 10% TCA was used as a blank; the extinction coefficient was 155 mM^−1^ cm^−1^. Ion leakage from the root and leaf was measured following the method by Zhao et al. [37] with minor modifications. Five root segments (around 1 cm long) and 10 leaf segments (around 0.5 cm diameter) were cut and rinsed three times with deionized water to remove the leakage ion due to the immediate injury inflicted on the tissue during cutting. The segments were then incubated in 10 mL vials containing 8 mL of deionized water for 3 h in a rotary shaker at room temperature. After being incubated for 3 h, the conductivity of the solution was measured, and it was then boiled at 100 °C for 30 min. After the solution was cooled to room temperature, the conductivity was measured again, and the results were expressed as relative conductivity [(conductivity after incubation/conductivity before boiling) × 100].

### 2.4. Antioxidant Enzyme Activity of the Rice Seedlings

To analyze the activity of the antioxidant enzyme in rice exposed to CuO NPs, 1 g of fresh roots and leaves were grounded in 10 mL precooled potassium phosphate buffer (PBS, 100 mM, pH 6.8) and then centrifuged for 20 min at 10,000 g. The supernatant was used to examine the activity of the antioxidant enzymes. The catalase (CAT, EC 1.11.1.6) was measured by monitoring the degradation of H_2_O_2_ at 240 nm, whose extinction coefficient was 39.4 mM^−1^ cm^−1^ [38], while the superoxide dismutase (SOD, EC 1.11.1.7) activity was quantified by monitoring the inhibition of nitroblue tetrazolium at 560 nm according to Beyer et al. [39]. The activity of guaiacol peroxidase (POD, EC 1.15.1.1) was detected by monitoring the formation of guaiacol dehydrogenation (extinction coefficient 6.39 mM^−1^ cm^−1^) at 420 nm [40]. The activities of CAT, SOD, and POD were performed with a Beckman Du-640 spectrometer (Beckman Coulter, Brea, CA, USA) at 25 °C.

### 2.5. Chlorophyll and Carotenoid Content

The content of chlorophyll and carotenoids in rice leaves was detected according to Nair and Chung [41]. Rice leaves with a weight of 0.05 g were incubated in 95% (*v*/*v*) ethanol (10 mL) for 3 days in the dark at 4 °C. After centrifugation, the absorption of the supernatant was measured by a Beckman Du-640 spectrometer (Beckman Coulter, Brea, CA, USA) at 470, 665, and 649 nm. Then, the content of chlorophyll and carotenoids was calculated according to Lichtenthaler and Wellburn [42].

### 2.6. Quantitative Real-Time PCR (qRT-PCR) of the Chlorophyll Synthesis Genes

The RNA was extracted using a TRIzol reagent (Bioteke, Beijing, China) following the manufacturer’s instructions. One microgram of the total RNA was reverse-transcribed into cDNA by a PrimeScript™ RT reagent kit (TaKaRa, Tokyo, Japan) according to the manufacturer’s protocol. Finally, qRT-PCR of four chlorophyll synthesis genes, namely, *Magnesium chelatase D subunit* (*CHLD*), *Chlorophyll synthase* (*CHLG*), *Magnesium-protoporphyrin IX methyltransferase* (*CHLM*), and *Chlorophyllide a oxygenase* (*CAO*), were performed. The *ACT2 (actin-2, LOC4349087)* [3] of rice plants was used as an internal reference. The primer pairs used in qRT-PCR analysis of *CHLD*, *CHLG*, *CHLM*, and *CAO* have been detailed in a previous study [33]. The qRT-PCR experiment was carried out with an Applied Biosystems 7500 Real-Time PCR System (Thermo Fisher Scientific, Waltham, MA, USA). Briefly, a total of 10 μL reaction volume was prepared with 0.2 μM forward and reverse primers, 5 μL SYBR Premix Ex Taq™ II (TaKaRa, Tokyo, Japan), and 1 μL template cDNA. The amplification program was as follows: initial denaturing at 95 °C for 5 min, 40 cycles of 10 s at 95 °C, 10 s at 60 °C, and 20 s at 72 °C. The fluorescence data were collected, and the relative expression levels of these genes were calculated with the formula 2^−^^△△Ct^ [43]. In the qRT-PCR analysis, the biological replicates were measured three times.

### 2.7. Statistical Analysis

The results are shown as mean ± standard deviation (SD). After the normality and homogeneity tests, the data were subjected to one-way ANOVA test in the SPSS software (Version 17.0) (SPSS, Chicago, IL, USA). Tukey’s honestly significant difference (HSD) multiple comparisons was utilized to conduct multiple comparisons. The significance level was *p* < 0.05.

## 3. Results and Discussion

### 3.1. Biomass Accumulation of Rice Seedlings

**Figure 1:**
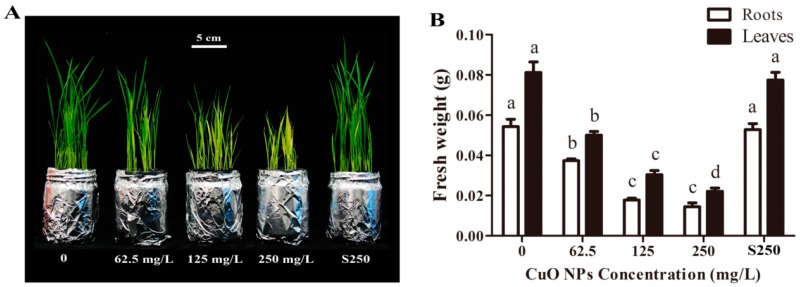
Effects of CuO NPs on the growth of rice seedlings. (**A**) Phenotype of rice seedlings; (**B**) Fresh weight of the rice roots and leaves exposed to 62.5, 125 and 250 mg/L CuO NPs, supernatant from 250 mg/L CuO NPs suspensions and without CuO NPs, for 7 days. The bars show the mean and SD of triplicate samples. Different letters represent significant differences between the means of the treatments (*p* < 0.05, Tukey-HSD).

After seven days, the seedlings exposed to CuO NPs showed phenotypic changes compared to the control plants (Figure 1A). Specifically, roots and shoots were shorter than those in the control, suggesting CuO NPs were toxic to rice plants, even at a low concentration of 62.5 mg/L. The statistical results showed that 62.5, 125, and 250 mg/L CuO NPs reduced the weight of rice roots by 31.1%, 67.2%, and 73.5%, respectively, compared to the control (*p* < 0.05) (Figure 1B). In addition, the weight of rice leaves treated with 62.5, 125, and 250 mg/L CuO NPs was 38.4%, 62.7%, and 72.8% lower, respectively, compared to the control (*p* < 0.05), suggesting that the phytotoxicity of CuO NPs in rice seedlings was dose-dependent. Indeed, such a dose-dependent manner of phytotoxicity has also been reported in various plants, e.g., *Schoenoplectus tabernaemontani* [27], *Hordeum vulgare* L. [28], wheat [44], and rice [30].

The toxicity of Cu^2+^ at a high level in plants has been reported previously [45,46]. It has been found that CuO NPs can release Cu ions in aqueous solutions [47,48], which may be a factor in causing the toxicity of CuO NPs in plants. In this study, the plants treated with supernatant from 250 mg/L CuO NP suspensions showed no observable phenotypic changes and changes in fresh weight of the rice seedlings compared to the control. Shi et al. [29] reported that CuO NP suspension could release 0.16 mg/L of Cu^2+^, which was not sufficient to lead to the phytotoxicity of CuO NPs in *Landoltia punctata*. Zhang et al. [27] also showed that the concentration of 0.06 mg/L, the level released from CuO NP suspension, did not affect the growth of *Schoenoplectus tabernaemontani*. Our previous study showed that the released Cu^2+^ (0.11 ± 0.04 mg·L^−1^) of 2000 mg/L CuO NPs showed no significant inhibition effects on the root growth of both maize and rice [32]. Therefore, these results suggest that the release of Cu^2+^ is probably not the main reason for the phytotoxicity caused by CuO NPs.

### 3.2. Integrity of the Cell Membrane System of Rice

**Figure 2:**
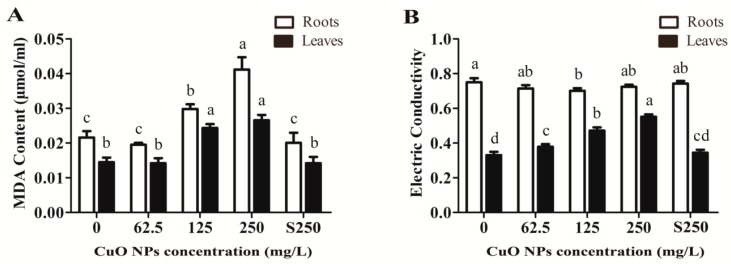
The effects of CuO NPs on the integrity of rice seedlings cells membrane system. (**A**) MDA content; (**B**) the electrical conductivity of rice seedlings exposed to 62.5, 125 and 250 mg/L CuO NPs, supernatant from 250 mg/L CuO NPs suspensions and have no CuO NPs for 7 days. The bars indicates the mean and SD of triplicate samples. Different letters represent significant differences between the means of treatments (*p* < 0.05, Tukey-HSD).

Under adverse conditions, plants can produce reactive oxygen species (ROS) that can cause damage to biological macromolecules and the cell membrane system [26]. As shown in Figure 2A, 125 and 250 mg/L CuO NPs significantly increased the MDA content of the rice shoots by 69.7% and 83.4%, respectively, compared to unexposed plants (control) (*p* < 0.05). Correspondingly, the levels of electrical conductivity in rice leaves treated with 62.5, 125, and 250 mg/L CuO NPs also increased by 14.6%, 42.7%, and 67.0%, respectively, compared to the control (*p* < 0.05) (Figure 2B). On the other hand, MDA content was relatively higher in rice roots exposed to 125 and 250 mg/L CuO NPs. Previous studies have also found decreases in plasma membrane integrity induced by CuO NPs in other plants, e.g., *Arabidopsis Thaliana* [49] and *Hordeum vulgare* L. [28]. Shaw and Hossain also reported that MDA content significantly increased in rice leaves exposed to CuO NPs for both 7 and 14 days [30]. Only 125 mg/L CuO NPs significantly reduced the electrical conductivity of rice roots by 6.5% (0.70 ± 0.01) compared to the control (0.75 ± 0.02), while the electrical conductivity showed unremarkable changes in rice roots exposed to 62.5 (0.72 ± 0.02) and 250 mg/L (0.72 ± 0.01) CuO NPs. Thus, it is supposed that rice roots may have probably generated a defense system to maintain ionic balance inside and outside the cells.

### 3.3. Antioxidant Enzyme Activity of Rice Seedlings

**Figure 3:**
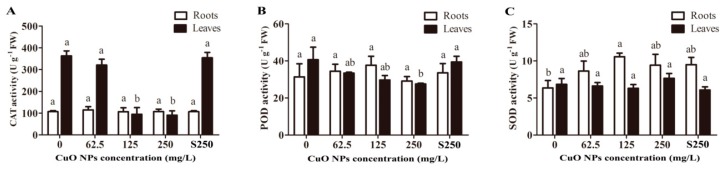
Effects of CuO NPs exposed to antioxidant enzyme activities. (**A**) CAT; (**B**) SOD, and (**C**) POD are the activities in the roots and shoots of rice seedlings exposed to 62.5, 125 and 250 mg/L CuO NPs, supernatant from 250 mg/L CuO NPs suspensions and without treatment for 7 days. The bars indicate the mean and SD of triplicate samples. Different letters represent significant differences between the means of treatments (*p* < 0.05, Tukey-HSD).

The integrity destruction of the rice cell membrane system was probably caused by increased reactive oxygen species under CuO NP treatment. As shown in Figure 3A, 125 and 250 mg/L CuO NPs significantly decreased the CAT activity of the rice leaves by 73.9% (94.84 ± 53.17) and 75.0% (90.71 ± 34.39), respectively, compared to the unexposed plants (363.18 ± 39.44) (*p* < 0.05). Only a high concentration of 250 mg/L CuO NPs significantly decreased the POD activity of the rice leaves by 32.3% (27.57 ± 0.69), while 62.5 (33.45 ± 0.85) and 125 mg/L (29.72 ± 4.13) CuO NPs showed no significant change compared to the control (Figure 3C), suggesting that rice leaves may have other active defense systems to protect them from ROS-mediated oxidative stress. Additionally, unremarkable changes were found in CAT and POD activities between the roots exposed to CuO NPs and the control roots (Figure 3A,C). SOD activity showed different trends to CAT and POD activity in rice seedlings. As shown in Figure 3B, the SOD activity was significantly upregulated by 166% (*p* < 0.05) in the rice roots treated with 125 mg/L CuO NPs, but no changes were observed in the rice leaves. Based on previous studies, many factors have been suggested as potential mechanisms of CuO NPs causing phytotoxicity, e.g., DNA damage, metal ions released from NPs, ROS generation, and oxidative stress [21]. Among them, oxidative stress has attracted considerable attention [28,30]. To protect themselves from the harm of excess H_2_O_2_, plants usually activate their defense system, including enzymatic (CAT, POD, etc.) and nonenzymatic (cytochrome *f*, proline, carotenoids, etc.) ways [50]. As important antioxidant enzymes, SOD can catalyze O^2−^ to H_2_O_2_, while CAT and POD can catalyze H_2_O_2_ to H_2_O [51]. The increasing SOD activity in the rice roots exposed to CuO NPs suggested that rice seedlings could generate significant amounts of ROS. However, there was no change in the activity of CAT and POD in rice roots exposed to CuO NPs. This result can probably be explained by the excess H_2_O_2_ existing in rice roots, which exceeded the maximum catalytic ability of CAT and POD.

### 3.4. Chlorophyll and Carotenoid Content of Rice Leaves

**Figure 4:**
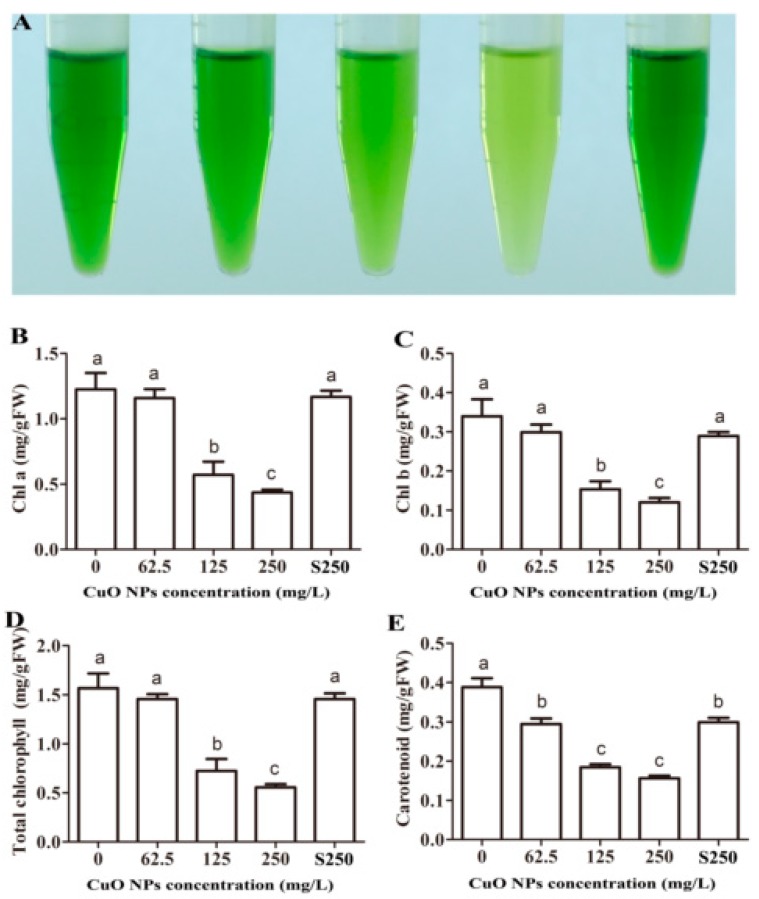
Effects of CuO NPs on the chlorophyll and carotenoid contents in rice leaves. (**A**) Photographs of chlorophyll extracted solution of 21-days-old rice leaves treated with 0, 62.5, 125, 250 mg/L CuO NPs, and supernatant from 250 mg/L CuO NPs (from left to right); (**B**) Chl-a contents; (**C**) Chl-b contents; (**D**) Total chlorophyll contents; (**E**) Carotenoid contents. The bars indicate the mean and SD of triplicate samples. Different letters represent significant differences between the means of the treatments (*p* < 0.05, Tukey-HSD).

The rice leaves exposed to increasing concentrations of CuO NPs gradually turned to yellow (Figure 1A). Chlorophyll and carotenoid extracts from exposed rice leaves also exhibited different colors compared to the control group and the plants treated with supernatant from 62.5 to 250 mg/L CuO NP suspensions (Figure 4A). The chlorophyll *a* (Chl-*a*) content in rice leaves treated with 125 and 250 mg/L CuO NPs decreased significantly by 53.5% and 70.4%, respectively, while the chlorophyll *b* (Chl-*b*) content was 54.8% and 64.7% lower, respectively, than the control (*p* < 0.05) (Figure 4B,C). The total chlorophyll content exhibited the same pattern as both Chl-*a* and Chl-*b* (Figure 4D). The carotenoid content decreased significantly by 24.1%, 52.5%, and 59.7% in rice leaves exposed to 62.5, 125, and 250 mg/L CuO NPs, respectively (*p* < 0.05) (Figure 4E). The photosynthetic yield and the content of Chl-*a* and Chl-*b* are considered important indicators in assessing the phytotoxicity of NPs, e.g., Ag NPs [52] and ZnO NPs [1,33]. Shaw et al. also reported that *Hordeum vulgare* L. with exposure to 0.5, 1.0, and 1.5 mM CuO NPs for 20 days had significantly decreased chlorophyll content [28]. Consistent with these reports, the decrease in chlorophyll content was also found in rice leaves exposed to CuO NPs in our study. Furthermore, photosynthesis in the rice seedlings was constrained, and plant growth was inhibited.

### 3.5. Synthesis of Chlorophyll and Carotenoid in Rice Leaves

**Figure 5:**
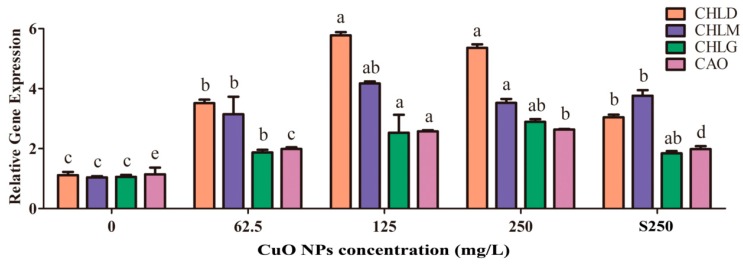
Effects of CuO NPs on the Chlorophyll synthesis genes in rice leaves treated with 0, 62.5, 125, 250 mg/L CuO NPs, or supernatant of 250 mg/L CuO NPs. The bars provide the mean and SD of triplicate samples. Different letters represent significant differences between the means of the treatments (*p* < 0.05, Tukey-HSD).

Four chlorophyll biosynthesis genes, namely, *CHLD*, *CHLG*, *CHLM*, and *CAO*, were found to be significantly upregulated according to different magnitudes of CuO NPs at 62.5, 125, and 250 mg/L. Specifically, 125 mg/L CuO NPs exhibited the maximum variation, with the level of *CHLD*, *CHLG*, *CHLM,* and *CAO* genes increasing by 5.18, 2.39, 4.02, and 2.25 times, respectively, compared to the control (*p* < 0.05) (Figure 5). Although the chlorophyll content in rice leaves treated with 250 mg/L CuO NPs was significantly less than those treated with 125 mg/L CuO NPs, the level of these four genes in plants treated with 250 mg/L CuO NPs was quite similar to that treated with 125 mg/L CuO NPs. In addition, the levels of *CHLD*, *CHLG*, *CHLM,* and *CAO* genes in plants treated with the supernatant of 250 mg/L CuO NPs also increased by 2.72, 1.74, 3.62, and 1.73 times compared to the control. Such a different pattern of chlorophyll content and expression level suggests that these rice seedlings exposed to CuO NPs could regulate the expression of some genes to adapt to stressed environment.

## 4. Conclusions

This study aimed to assess the effects of CuO NPs on rice seedlings by estimating phenotypic changes and the relevant physiological responses. The results indicated that 62.5, 125, and 250 mg/L CuO NPs suspended in Yoshida nutrient solution could significantly inhibit the growth of rice seedlings and chlorophyll content. The oxidative damage was also seen in rice shoots exposed to CuO NPs, and the MDA content and electrical conductivity were significantly upregulated compared to the control. The activity of SOD was also greater in rice roots exposed to 125 mg/L CuO NPs. The content of chlorophyll, including Chl-*a* and Chl-*b*, and carotenoids decreased, while four chlorophyll synthesis genes, namely, *CHLD*, *CHLG*, *CHLM*, and *CAO*, significantly increased. Overall, the findings in this study could provide evidence for risk assessment and guide applications of CuO NPs in agriculture. However, the toxicity in rice seedlings was obtained by short exposure to high levels of CuO NPs, which may not exist in the natural environment. The potential impacts of long-term exposure to CuO NPs at low concentrations on edible plants and their bioaccumulation in edible portions need to be investigated in further research.
